# Laser treatment of hypertrophic scars: the operative and peri-operative practices of burns clinicians

**DOI:** 10.1007/s10103-026-04919-z

**Published:** 2026-06-24

**Authors:** Maria Shilova, Roy Kimble, Robert S Ware, Karin Plummer, Orlando Flores, Hui Grace Xu, Bronwyn Griffin

**Affiliations:** 1 School of Nursing and Midwifery, Griffith University, Brisbane, Australia; 2 Centre for Children’s Burns and Trauma Research, Children’s Health Queensland Hospital and Health Service, Brisbane, Australia; 3 Paediatric Surgery, Urology, Burns and Trauma, Queensland Children’s Hospital, Brisbane, Australia; 4 Griffith Biostatistics Unit, Griffith Health, Griffith University, Brisbane, Australia; 5 Wesley Research Institute, Brisbane, Australia; 6 Research Department, COANIQUEM, Santiago, Chile; 7 School of Nursing, Queensland University of Technology, Brisbane, Australia; 8 Nursing & Midwifery Research Centre, Royal Brisbane and Women’s Hospital, Brisbane, Australia

**Keywords:** Hypertrophic scars, Scars, Keloids, Laser, Questionnaire

## Abstract

Purpose. Laser treatment is rapidly becoming a staple of hypertrophic scar management, but literature reviews have identified significant heterogeneity in laser protocols. Mapping laser operative practices can help to share clinical expertise and identify new research directions. A questionnaire to characterize laser treatment techniques for hypertrophic scars was conducted. Methods. The questionnaire was developed with a multidisciplinary team of burns clinicians and researchers and translated into Spanish, Chinese and German. It was distributed internationally to burns clinicians between December 2021 and June 2023. Results. Forty-three clinicians participated in the questionnaire. Respondents used laser to treat both immature (n = 36, 84%) and mature (n = 42, 98%) scars, with varying approaches to how soon therapy should commence post-injury, and how frequently it should be repeated. Almost all respondents used CO_2_ fractional ablative laser (n = 41, 95%) for scar treatment, one-third used pulsed dye laser (n = 14, 33%), and approximately one-half reported using more than one laser during a single procedure (n = 21, 49%). Respondents used scar thickness to assist with setting laser parameters (n = 29, 67%), chiefly determining this by estimation (n = 25, 58%). 60% of respondents performed scar reconstruction (n = 26), and 91% (n = 39) used adjunct therapies, such as corticosteroids, concurrently with laser treatment. Choices of anesthetic, analgesia and dressings varied widely. Conclusion. While there are similarities in the broad aspects of how burns clinicians use laser to treat hypertrophic scars, there are several differences in practice. These variations highlight potential research directions to optimize patient outcomes from this treatment modality.

**Supplementary Information:**

The online version contains supplementary material available at 10.1007/s10103-026-04919-z.

## Introduction

Laser treatment is an increasingly popular hypertrophic scar (HTS) management option [1] that improves scar characteristics [2], function [3], appearance [2] and quality of life [4]. However, exactly how to best use laser technology to treat HTSs is unclear. Systematic reviews have identified that a variety of laser settings [3, 5], treatment session frequencies [6], and laser-assisted drug delivery techniques are used for the treatment of HTSs [7]. An expert consensus guideline for the laser treatment of traumatic scars has been published, but is not supported by high-quality, rigorous research [8]. However, there is clinical interest in conducting such research and developing an evidence base [8].

Developing an evidence base for laser use is essential, and this goal can be assisted by investigating how clinicians currently use laser technology for HTS treatment. We conducted an international questionnaire study on the laser treatment techniques for HTSs, with the aim of characterizing current practices to share clinical expertise and provide directions for research.

## Methods

A descriptive survey of burns clinicians who use laser technology to treat HTSs was conducted from December 2021 to June 2023, delivered through an online questionnaire. The questions focused on pre-procedural, intra-procedural and post-procedural care. This study received ethical review exemption from the Queensland Children's Hospital Human Research Ethics Committee (EX/21/QCHQ/79011) and approval from the Griffith University Human Research Ethics Committee (2021/828). 

The questions were designed with two consultant pediatric burns surgeons with more than 40 combined years of clinical and burn research experience, a burns nurse practitioner with 26 years of clinical experience, and the burns research team conducting the study. The majority of the authors are based at a quaternary pediatric burns center with laser treatment capacity since 2019.

The questions addressed aspects of the laser scar treatment procedure and peri-procedural care, based on the experiences of the clinicians, laser scar treatment methodologies reported in literature and known literature gaps [7–13]. The final base set of 12 questions and further clarifying questions (displayed depending on answers given by the respondent, see Supplementary Material 1) was delivered via the Griffith Online Research Survey Tool platform (Griffith University, Australia). The English questionnaire was translated by burns researchers whose first language was Spanish, Chinese and German. To confirm that concepts and questions were communicated equivalently in the questionnaire versions, forward and backward translation was performed, and discussed among the research team and translators.

Potential participants were identified via existing professional clinical and research networks and were emailed with an invitation to participate in the study. The questionnaire was also distributed at conferences (Australian and New Zealand Burn Association Annual Scientific Meeting, the American Burn Association Annual Meeting, the European Burns Association Congress and the International Society for Burns Injuries Congress), and via the American Burn Association mailing list (see Supplementary Material 2). Participating clinicians were asked to forward the questionnaire to any colleagues who also use laser to treat HTSs, using a snowball recruitment strategy.

Participants provided informed consent to participate in the study at the beginning of the questionnaire. Identifying data were collected to avoid invitation and response duplication. See Supplementary Material 3 for further methodological details described according to the Checklist for Reporting Results of Internet E-Surveys [14].

### Data analysis

Summary statistics are presented as frequency and percentage for categorical variables. Content analysis of descriptor responses was undertaken, and responses were categorized together where appropriate (e.g. “Kenacort” and “triamcinolone”). Data were summarized in Microsoft Excel (Version 2312, Microsoft Corporation, Washington, United States).

## Results

### Participant characteristics

There were 43 responses to the questionnaire (excluding three duplicates), predominantly in English (*n* = 39, 91%), from clinicians in Africa, Asia, Australia, Europe, North America and South America. Most respondents were surgeons, who treated both children and adults (Table 1).

**Table 1:** Demographics of respondents, indications for scar treatment and laser scar treatment techniques

Question set	Characteristic	*n* (%)
Profession of respondent	Surgeon	38 (89%)
	Plastic surgeon	14 (33%)
	Burns surgeon	12 (28%)
	Pediatric surgeon	5 (12%)
	Surgeon (unspecified sub-specialty)	7 (16%)
	Dermatologist	2 (5%)
	Skin therapist	1 (2%)
	Pediatrician	1 (2%)
Population treated	Children	12 (28%)
	Adults	8 (19%)
	Both children and adults	23 (53%)
Scars treated	Immature	36 (84%)
	Mature	42 (98%)
Timing of first laser procedure post-injury	< 1 month	0 (0%)
	1–3 months	15 (41%)
	3–6 months	11 (30%)
	6–12 months	6 (16%)
	> 12 months	2 (5%)
	Other^a^	3 (8%)
Scar characteristics considered as indications for laser treatment^b^	Scar thickness	15 (88%)
	Poor pliability	13 (76%)
	Pain	12 (71%)
	Pruritus	15 (88%)
	Dyschromia	6 (35%)
	Decreased range of motion	1 (6%)
Type of laser used for scar treatment	CO_2_ fractional ablative laser	41 (95%)
	Er: YAG fractional ablative laser	1 (2%)
	Pulsed dye laser	14 (33%)
	Intense pulsed light	3 (7%)
	Low-level biostimulatory laser	1 (2%)
Use of scar thickness to guide laser parameter selection	Scar thickness used to guide settings	29 (67%)
	Scar thickness on clinical examination	25 (58%)
	Scar thickness measured by a measuring device (e.g. calipers)	4 (9%)
	Scar thickness measured by ultrasound	6 (14%)
	Scar thickness not used to guide settings	14 (33%)
Adjunct therapies during laser	Topical corticosteroids	18 (42%)
	Intralesional corticosteroids	2 (5%)
	Both topical and intralesional corticosteroids	5 (12%)
	Corticosteroids delivered by unspecified route	12 (28%)
	Contact therapy	1 (2%)
	Compression therapy	1 (2%)
	Topical platelet-rich plasma	1 (2%)
	Chemical peeling with 5% retinoic acid cream	1 (2%)
Intervals between laser procedures^d^	< 1 months	0 (0%)
	1–3 months	31 (74%)
	> 3 months	6 (14%)
	Other^c^	5 (12%)
Number of procedures required to achieve a satisfactory outcome^b^	1	0 (0%)
	2–5	12 (71%)
	> 5	5 (29%)
Factors determining need for further laser treatment^b, e^	Improvement from first procedure	15 (88%)
	Patient satisfaction with outcome of first procedure	14 (82%)
	Scar characteristics	10 (59%)
	Relief of scar symptoms (pain, pruritus) from first procedure	3 (18%)
	Wound result from first procedure	2 (12%)
	Patient perception of improvement from first procedure	3 (18%)

a: “other” timeframes specified in free text comments were: (1) > 6 weeks after formation of stable epithelium, (2) one month after wound closure, (3) almost all of the provided options. 6 participants did not provide a response to this question

b: this question was added when the questionnaire was distributed via the American Burn Association mailing list, where there were 17 respondents. The percentage is calculated as a proportion of this group, not the total number of questionnaire responders

c: “other” timeframes specified in free text comments were: (1) dependent on secondary factors, (2) 6–8 weeks for two respondents, (3) 3 months for two respondents

d: one participant left this question blank, hence percentages are calculated as a proportion of 42 respondents, not 43

e: some of the listed factors were extracted from free text comments made by respondents

### Laser procedure

All respondents believed that laser treatment was best commenced at least a month after the inciting injury (Table 1), with 1–3 months being the most common timeframe for the first laser procedure (*n =* 15, 41%). Practitioners considered a range of scar characteristics to be important when deciding if a patient’s HTS should be treated with laser (Table 1).

The most commonly used device (*n* = 41, 95%) was a CO_2_ fractional ablative laser, although almost half of the respondents (*n =* 21, 49%) used more than one type of laser during a single procedure. When setting laser parameters, scar thickness was used to guide selection by 67% of participants (*n =* 29, Table 1), most frequently estimated by clinical examination (*n =* 25, 58%).

Many respondents combined the laser procedure with adjunct therapies (*n* = 39, 91%) or surgical scar reconstruction (*n* = 26, 60%). Using corticosteroids in some form was the most common adjunct treatment (*n* = 37, 86%), although not all participants specified the route of delivery (Table 1). 42% of participants (*n =* 18) specified applying topical corticosteroids after laser, and listed various formulations for this, including cream (*n =* 4, 9%) and ointment (*n =* 2, 5%). Free text comments listed triamcinolone, hydrocortisone and dexamethasone as being specific forms of corticosteroids used together with laser therapy.

**Fig. 1:**
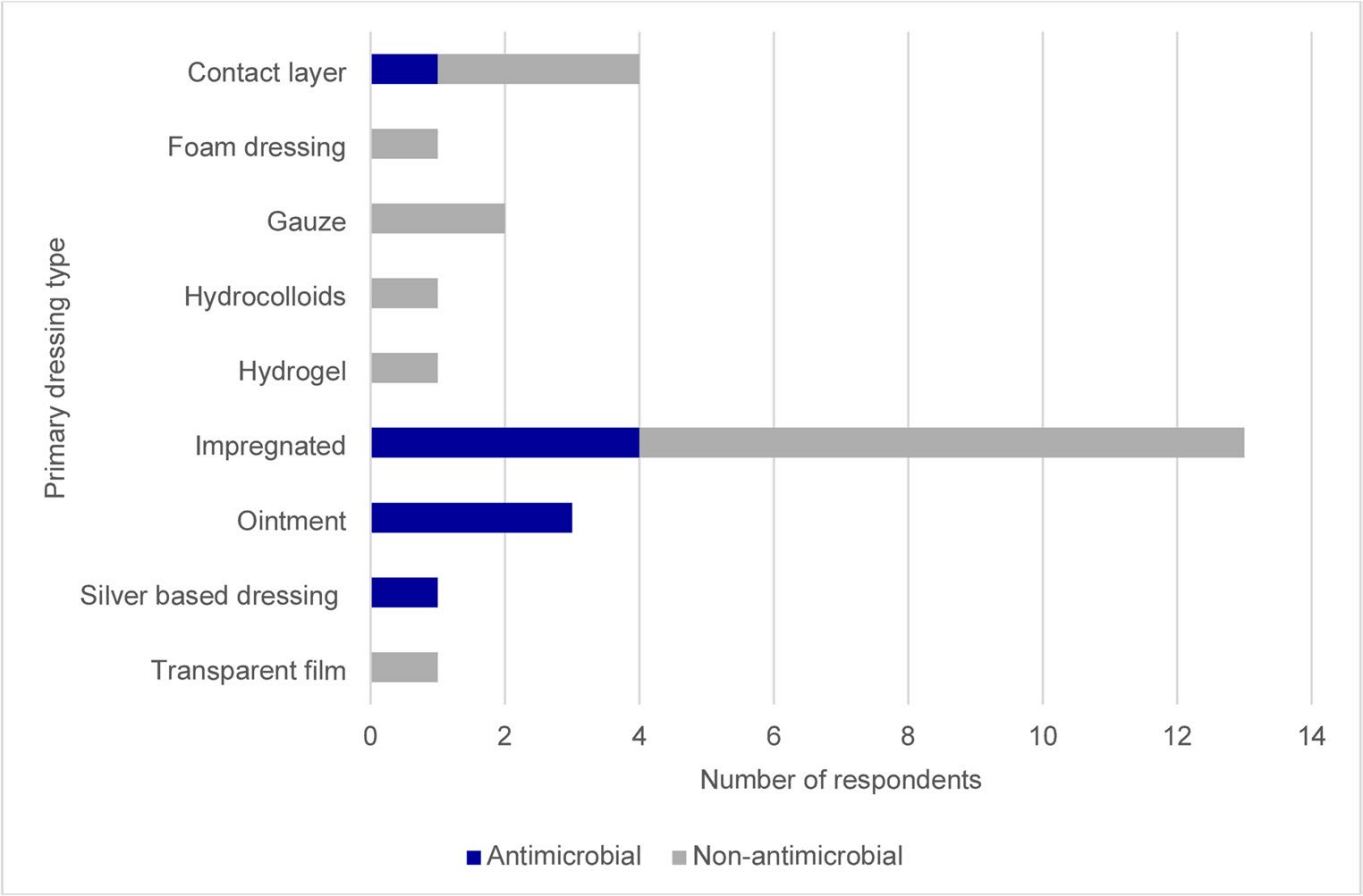
Primary dressings for post-laser treatment of hypertrophic scars. This chart illustrates the primary dressing choices for post-laser procedures for hypertrophic scars. The dressing types have been classified according to the Centers for Medicare and Medicaid Services classification [15, 16]. Impregnated dressings, such as Bactigras™ (Smith and Nephew, London UK), Xeroform™ (Covidien, Mansfield USA) and Adaptic™ (Systagenix, North Yorkshire UK) were the most common primary dressing choice, covered with a dry or adhesive dressing. Two respondents provided inadequate detail of the dressing type; these were not included in this figure. One clinician left this question blank, so was not included in this analysis

Post-procedurally, 71% of clinicians (*n =* 30) used a dressing, although the type of dressing varied (Fig. 1), with further variation in brands of dressing used. When evaluating if further procedures were required, clinicians considered several patient and scar factors (Table 1) and indicated that 2–5 procedures (*n =* 12, 71%) were most often needed to achieve a good clinical outcome. Such procedures were typically performed 1–3 months apart (*n =* 31, 74%, Table 1). 

### Anesthesia and analgesia for laser procedures

Almost all respondents (*n =* 42, 98%) used anesthetic (either general or local) for laser procedures (see Table 2). Post-operative analgesic choices varied between respondents (Table 2), with non-steroidal anti-inflammatory agents being favored as an analgesic for post-laser pain (*n* = 32, 74%).

**Table 2:** Anesthesia and post-operative analgesia for laser treatment of hypertrophic scars. Multiple responses were allowed for the post-operative analgesia question

Stage of procedure	Anesthesia/analgesia used	*n* (%)
Intraoperative	Local anesthetic only	23 (55%)
	General anesthetic only	11 (26%)
	Both local and general anesthetic	5 (12%)
	Sedation	3 (7%)
Post-operative	None	4 (9%)
	Ice-pack or frozen topics	2 (4%)
	Topical lidocaine	1 (2%)
	Paracetamol	9 (21%)
	Non-steroidal anti-inflammatory medications	32 (74%)
	Opioid medications	6 (14%)
	Neuropathic pain mediations	6 (14%)

## Discussion

This questionnaire identified several points of practice variation for the laser treatment of HTSs which could benefit from targeted research. The first of these variations was the use of adjunct therapies. Most respondents used corticosteroids during laser treatment, but routes of delivery and the exact agents used varied. While there have been modestly-sized trials investigating if using corticosteroids concurrently with laser treatment improves patient outcomes [7, 17–20], their results have not been congruent with one another, and no trials have compared different corticosteroid vehicles (e.g. ointment, cream, suspension). Multi-institution collaboration could produce larger trials of this adjunct therapy and investigate the effect of different routes and formulations of corticosteroid delivery.

The choice of anesthetic and post-operative analgesia was also heterogeneous, in line with clinician surveys on fractional ablative laser resurfacing [21] and pulsed dye laser treatment [22]. This variation is not surprising, as analgesic and anesthetic choice is determined by patient, lesion and procedure characteristics [23]. Investigation of which specific factors guided clinicians’ anesthetic and analgesic regimens for laser procedures was outside the scope of this questionnaire. However, it is important to investigate analgesia and anesthesia for laser procedures in future, as some agents may be more effective or appropriate in this setting. For example, Edkins et al., found that using topical anesthesia for patients undergoing laser treatment for burns HTS under general anesthetic reduced intraprocedural opioid use and decreased time to discharge post-operatively [24]. Further, similar studies could inform anesthetic and analgesic choices for patients with HTS undergoing laser treatment to optimize patient comfort and optimize medical resource use.

Post-operative dressing choices also varied widely among our participants, similar to the findings of a clinician survey on post-laser resurfacing care [25]. The relationship of dressings with patient outcomes and adverse reactions (such as the presence of persistent pixel marks or a hypertrophic scar reaction post-laser), has not been investigated and could be the subject of future research.

## Limitations

The questionnaire intends to provide a snapshot of practice, rather than a comprehensive representation of all practice worldwide, and has some associated limitations. Firstly, the questionnaire was primarily completed by English-speaking surgeons, which could limit the generalizability of the results to other clinician populations. Secondly, due to snowball recruitment being used to increase the number of responses, a response rate could not be obtained. Third, due to needing to keep the questionnaire length acceptable to respondents, we could not include questions on all aspects of laser HTS treatment. This left some unanswered questions, such what factors influence anesthetic and post-operative analgesic choices. Future questionnaires or semi-structured interviews could investigate specific aspects of practice in more detail. Finally, the phrasing of some questions was kept open to allow participants to include previously unidentified practices in their response. However, this resulted in a mixed level of detail within the responses. One example of this was the question on adjunct therapies, where some respondents wrote which formulation of corticosteroid they used and by what route it was delivered, and whilst some wrote less detailed responses. This limited our capacity to present data related to what exact corticosteroids are used and by what route. However, it did identify a point of practice heterogeneity to be investigated in future.

## Conclusion

This questionnaire investigated how laser is used to treat HTSs specifically, which has not been the focus of other questionnaires on laser operative practices [21, 22, 25]. The variation identified in practices offers the opportunity for international collaborative research to clarify which laser protocols work best for which scars, to learn from international clinical expertise and improve patient outcomes.

## Supplementary Information

Below is the link to the electronic supplementary material.

Supplementary Material 1

Supplementary Material 2

Supplementary Material 3

## Data Availability

Raw data from this study are not publicly available, as they contain re-identifiable elements, and participant informed consent did not extend to the use of the data for other studies. The manuscript contains the de-identified and analyzed data generated in this study.

## References

[1] Alexiades M (2025) Introduction 2025 special dermatology plastic surgery issue. Lasers Surg Med 57(1):7. 10.1002/lsm.23874 39878547 10.1002/lsm.23874

[2] Buhalog B, Moustafa F, Arkin L, Lee K, Siwy K, Donelan M, Hultman CS, Shumaker PR (2021) Ablative fractional laser treatment of hypertrophic burn and traumatic scars: a systematic review of the literature. Arch Dermatol Res 313(5):301–317. 10.1007/s00403-020-02135-5 32926192 10.1007/s00403-020-02135-5

[3] Pham VX, Pousti BT, Gauglitz G, Shumaker PR (2025) Impact of fractional ablative laser therapy on function, symptoms, and quality of life in the management of traumatic scars: a review. Lasers Surg Med 57(1):8–14. 10.1002/lsm.23858 39523456 10.1002/lsm.23858

[4] Liu H, Shu F, Xu H, Ji C, Wang Y, Lou X, Luo P, Xiao S, Xia Z, Lv K (2022) Ablative fractional carbon dioxide laser improves quality of life in patients with extensive burn scars: a nested case–control study. Lasers Surg Med 54(9):1207–1216. 10.1002/lsm.23603 36116066 10.1002/lsm.23603

[5] Li P, Zhang Q, Tao C, Liang H, Li D (2025) Pulsed dye laser, fractional CO_2_ laser, or combination for burn scar treatment: a systematic review. Lasers Med Sci 40:389. 10.1007/s10103-025-04629-y 41003790 10.1007/s10103-025-04629-y

[6] Pang X, Chi H, Zhan Z, Yu Z, Cai M (2024) CO(2) laser treatment for scars after cleft lip surgery: a systematic review and meta-analysis. BMC Oral Health 24(1):1443. 10.1186/s12903-024-05205-6 39604962 10.1186/s12903-024-05205-6 PMC11603913

[7] Shilova M, Plummer K, Ware R, Kimble R, Clark J, Cho E, McMillan L, Kimble L, Meikle B, Kunde L, Griffin B (2026) Laser-assisted drug delivery for hypertrophic scar treatment: a scoping review. J Burn Care Res 47(1):130–146. 10.1093/jbcr/iraf167 40971504 10.1093/jbcr/iraf167 PMC12770983

[8] Seago M, Shumaker PR, Spring LK et al (2020) Laser treatment of traumatic scars and contractures: 2020 international consensus recommendations. Lasers Surg Med 52(2):96–116. 10.1002/lsm.23201 31820478 10.1002/lsm.23201

[9] Kauvar ANB, Kubicki SL, Suggs AK, Friedman PM (2020) Laser therapy of traumatic and surgical scars and an algorithm for their treatment. Lasers Surg Med 52(2):125–136. 10.1002/lsm.23171 31621930 10.1002/lsm.23171

[10] Kent RA, Shupp J, Fernandez S, Prindeze N, DeKlotz CMC (2020) Effectiveness of early laser treatment in surgical scar minimization: a systematic review and meta-analysis. Dermatol Surg 46(3):402–410. 10.1097/dss.0000000000001887 30893164 10.1097/DSS.0000000000001887

[11] Issler-Fisher AC, Fisher OM, Haertsch P, Li Z, Maitz PKM (2020) Ablative fractional resurfacing with laser-facilitated steroid delivery for burn scar management: does the depth of laser penetration matter? Lasers Surg Med 52(2):149–158. 10.1002/lsm.23166 31571242 10.1002/lsm.23166

[12] Żądkowski T, Nachulewicz P, Mazgaj M, Woźniak M, Cielecki C, Wieczorek AP, Beń-Skowronek I (2016) A new CO2 laser technique for the treatment of pediatric hypertrophic burn scars: an observational study. Medicine 95(42):e5168. 10.1097/md.0000000000005168 27759650 10.1097/MD.0000000000005168 PMC5079334

[13] Mirmanesh M, Borab Z, Gantz M, Maguina P (2017) Peri-procedure laser scar therapy protocol: a pilot survey of plastic surgeons’ practices. Aesthetic Plast Surg 41(3):689–694. 10.1007/s00266-017-0862-7 28374298 10.1007/s00266-017-0862-7

[14] Eysenbach G (2004) Improving the quality of Web surveys: the Checklist for Reporting Results of Internet E-Surveys (CHERRIES). J Med Internet Res 6(3):e34. 10.2196/jmir.6.3.e34 15471760 10.2196/jmir.6.3.e34 PMC1550605

[15] Irion GL, Gardner JA, Pignataro RM (2024) Ch16 Selection and application of dressings. Comprehensive wound management, 3rd edn. CRC, Boca Raton, pp 297–328

[16] US Centers for Medicare & Medicaid Services (2024) Surgical Dressings - Policy Article. https://www.cms.gov/medicare-coverage-database/view/article.aspx?articleid=54563&ver=54&keyword=DME&keywordType=starts&areaId=s53&docType=NCA%2CCAL%2CNCD%2CMEDCAC%2CTA%2CMCD%2C6%2C3%2C5%2C1%2CF%2CP&contractOption=all&sortBy=relevance&bc=1. Accessed 4 November 2025

[17] Goel J, Singh VK, Haq A, Pp S, Verma S (2025) Split-scar technique to assess the efficacy of Er:YAG laser with intralesional triamcinolone combination on post-burn scars - A double blind, parallel, two-arm randomized controlled trial. Burns 51(8):107644. 10.1016/j.burns.2025.107644 40834485 10.1016/j.burns.2025.107644

[18] Manuskiatti W, Kaewkes A, Yan C, Ng JN, Glahn JZ, Wanitphakdeedecha R (2021) Hypertrophic scar outcomes in fractional laser monotherapy versus fractional laser-assisted topical corticosteroid delivery: a randomized clinical trial. Acta Derm Venereol 101(3):adv00416. 10.2340/00015555-3781 33686446 10.2340/00015555-3781 PMC9366502

[19] Lin CH, Tsai YJ, Chi SY, Hsieh MH, Lin KC, Lin HP, Hsu SY, Tsai HH, Hsieh CH (2022) Facilitated delivery of topical steroids after fractional ablative carbon dioxide laser benefits postthyroidectomy hypertrophic scar. Dermatologica Sinica 40(1). 10.4103/ds.ds_54_21

[20] Lin K-C, Wu S-C, Chi S-Y, Lin H-P, Lin C-H, Tsai Y-J, Hsieh M-H, Hsu S-Y, Hsieh C-H (2021) Facilitated delivery of topical steroids after fractional ablative carbon dioxide laser failed to prevent the postthyroidectomy hypertrophic scar. Dermatologica Sinica 39(3):118–124. 10.4103/ds.ds_29_21

[21] Gold M, Andriessen A, Cohen JL, Goldberg DJ, Grover K, Hu S, Mandy SH, Vega JMK (2020) Pre-/postprocedure measures for laser/energy treatments: A survey. J Cosmet Dermatol 19(2):289–295. 10.1111/jocd.13259 31840388 10.1111/jocd.13259

[22] Loh TY, Cotton CH, Vasic JB, Goldberg GN (2021) Current practices in pediatric dermatology laser therapy: an international survey. Lasers Surg Med 53(7):946–952. 10.1002/lsm.23327 32956533 10.1002/lsm.23327

[23] Yates B, Whalen J, Makkar H (2017) An age-based approach to dermatologic surgery: Kids are not just little people. Clin Dermatol 35(6):512–516. 10.1016/j.clindermatol.2017.09.017 29191343 10.1016/j.clindermatol.2017.09.017

[24] Edkins RE, Hultman CS, Collins P, Cairns B, Hanson M, Carman M (2015) Improving comfort and throughput for patients undergoing fractionated laser ablation of symptomatic burn scars. Ann Plast Surg 74(3):293–299. 10.1097/sap.0000000000000367 25664406 10.1097/SAP.0000000000000367

[25] Duke D, Grevelink JM (1998) Care before and after laser skin resurfacing. A survey and review of the literature. Dermatol Surg 24(2):201–206. 10.1111/j.1524-4725.1998.tb04138.x 9491114 10.1111/j.1524-4725.1998.tb04138.x

