# Supplementary material for: Laser treatment of hypertrophic scars: the operative and peri-operative practices of burns clinicians
Source: Lasers Med Sci. 2026 Jun 24;41(1):128. doi: 10.1007/s10103-026-04919-z (PMC13294306; doi:10.1007/s10103-026-04919-z)
Supplement: Supplementary file 1 — Supplementary Material 1 [file 10103_2026_4919_MOESM1_ESM.pdf]

Article title: *Laser treatment of hypertrophic scars: the operative and peri-operative practices of burns clinicians*

Journal title: Lasers in Medical Science

Authors: Maria Shilova, Roy Kimble, Robert S Ware, Karin Plummer, Orlando Flores, Hui (Grace) Xu, Bronwyn Griffin.

Corresponding author: Maria Shilova (School of Nursing and Midwifery, Griffith University; Children's Health Queensland, South Brisbane; Centre for Children's Burns and Trauma Research, Children's Health Queensland, South Brisbane, maria.shilova@griffithuni.edu.au)

Supplementary Material 1

This table lists the questions included in the questionnaire, and answer options (where the question was multiple choice).

Questions in all questionnaires

Question number	Question	Answer type
1	Do you treat adults/children/both?	Multiple choice (single selection allowed):<br>• Adults<br>• Children<br>• Both
2	What scars do you use lasers for?	Multiple choice (multiple selections allowed):<br>• Immature hypertrophic scars<br>• Mature hypertrophic scars
3	How long do you wait post-injury prior to commencing laser procedures on the area?	Multiple choice (multiple selections allowed):<br>• <1 month<br>• 1-3 months<br>• 3-6 months<br>• 6-12 months<br>• >12 months<br>• Other (free text box)
4	Do you estimate scar thickness before the procedure to help select laser parameters?	Yes/No
4a	Displayed if "yes" selected in answer to question 4. How do you do this?	Multiple choice (multiple selections allowed):<br>• Estimate thickness based on clinical examination<br>• Measure using a ruler or similar measuring device<br>• Using ultrasound<br>• Other (free text box)
5	Which lasers do you use?	Multiple choice (multiple selections allowed):<br>• CO_2 fractional laser<br>• Er:YAG fractional laser<br>• Pulsed dye laser<br>• Other (free text box)

6	Do you sometimes use more than one type of laser during procedures?	Yes/No answer
7	Do you use any form of local or general anesthesia during laser procedures?	Yes/No answer
7a	Displayed if "yes" selected in answer to question 7. What type of anesthesia do you use?	Multiple choice (one selection allowed):<br>• Local anesthetic<br>• General anesthetic<br>• Both local and general anesthetic<br>• Other (free text box)
8	Some centers use complementary therapies with laser procedures (e.g. applying topical steroids onto the scar shortly after the laser has been used on the scar). Do you use any such complementary therapies?	Yes/No answer
8a	Displayed if "yes" selected in answer to question 8. What complementary therapies do you use?	Free text box
9	Do you perform any other procedures at the same time as laser treatment for hypertrophic scars?	Yes/No answer
9a	Displayed if "yes" selected in answer to question 9. What type of procedures do you do?	Multiple choice (multiple selections allowed):<br>• Surgical reconstruction<br>• Other (free text box)
10	Are dressings used following laser procedures at your hospital?	Yes/No answer
10a	Displayed if "yes" selected in answer to question 10.<br>Please detail dressing types and for how long they are applied after the procedure.	Free text
11	What analgesia works best for pain following laser procedures, in your experience?	Multiple choice (multiple selections allowed):<br>• Non-steroidal anti-inflammatory medications<br>• Opioid medications<br>• Neuropathic pain medications (e.g. gabapentin)<br>• Other (free text box)
12	If repeat laser procedures are required, what is the typical time frame between these?	Multiple choice (one selection allowed):<br>• <1 month<br>• 1-3 months<br>• >3 months<br>• Other (free text box)

Questions added to questionnaire distributed via the American Burn Association

As per feedback from the American Burn Association (ABA), a further four questions were added to the questionnaire.

Question number	Question	Answer type
S1	What is the most common reason you suggest using laser to treat scars?	Multiple choice (multiple selections allowed):<br>• Scar thickness<br>• Poor scar pliability

		• Pain associated with the scar<br>• Pruritic associated with the scar<br>• Scar dyschromia<br>• Other (free text box)
S2	What is the typical number of laser procedures patients need to achieve a good clinical outcome?	Multiple choice (single selection allowed):<br>• 1<br>• 2-5<br>• >5<br>• Other:
S2a	Displayed if any other than "1" is selected in answer to question S2.<br>What factors play a role in determining if patients need repeat laser procedures?	Multiple choice (multiple selections allowed, with text boxes for each to allow for additional comments)<br>• The effect from the first procedure<br>• Patient satisfaction with the outcome<br>• Scar features<br>• Other
S3	How are laser procedures funded in your hospital/center?	Multiple choice (multiple selections allowed):<br>• Entirely public funding<br>• Entirely private funding<br>• Other (free text box)
S4	What barriers might your patients face in getting laser treatment?	Free text
