# Supplementary material for: Laser treatment of hypertrophic scars: the operative and peri-operative practices of burns clinicians
Source: Lasers Med Sci. 2026 Jun 24;41(1):128. doi: 10.1007/s10103-026-04919-z (PMC13294306; doi:10.1007/s10103-026-04919-z)

Article title: *Laser treatment of hypertrophic scars: the operative and peri-operative practices of burns clinicians*

Journal title: Lasers in Medical Science

Authors: Maria Shilova, Roy Kimble, Robert S Ware, Karin Plummer, Orlando Flores, Hui (Grace) Xu, Bronwyn Griffin.

Corresponding author: Maria Shilova (School of Nursing and Midwifery, Griffith University; Children's Health Queensland, South Brisbane; Centre for Children's Burns and Trauma Research, Children's Health Queensland, South Brisbane, maria.shilova@griffithuni.edu.au)

Supplementary Material 2

This figure describes the method of questionnaire development and distribution. The questionnaire was developed with a multidisciplinary burn clinical and research team and piloted for question clarity, clinical relevance and length by clinicians. The final questionnaire was translated into Spanish, Chinese and German by burns researchers who were native speakers in the languages. The survey was distributed at burns conferences and via email to head clinicians of burns units with further snowball recruitment through these avenues. The English survey was also distributed by the American Burn Association mailing list.

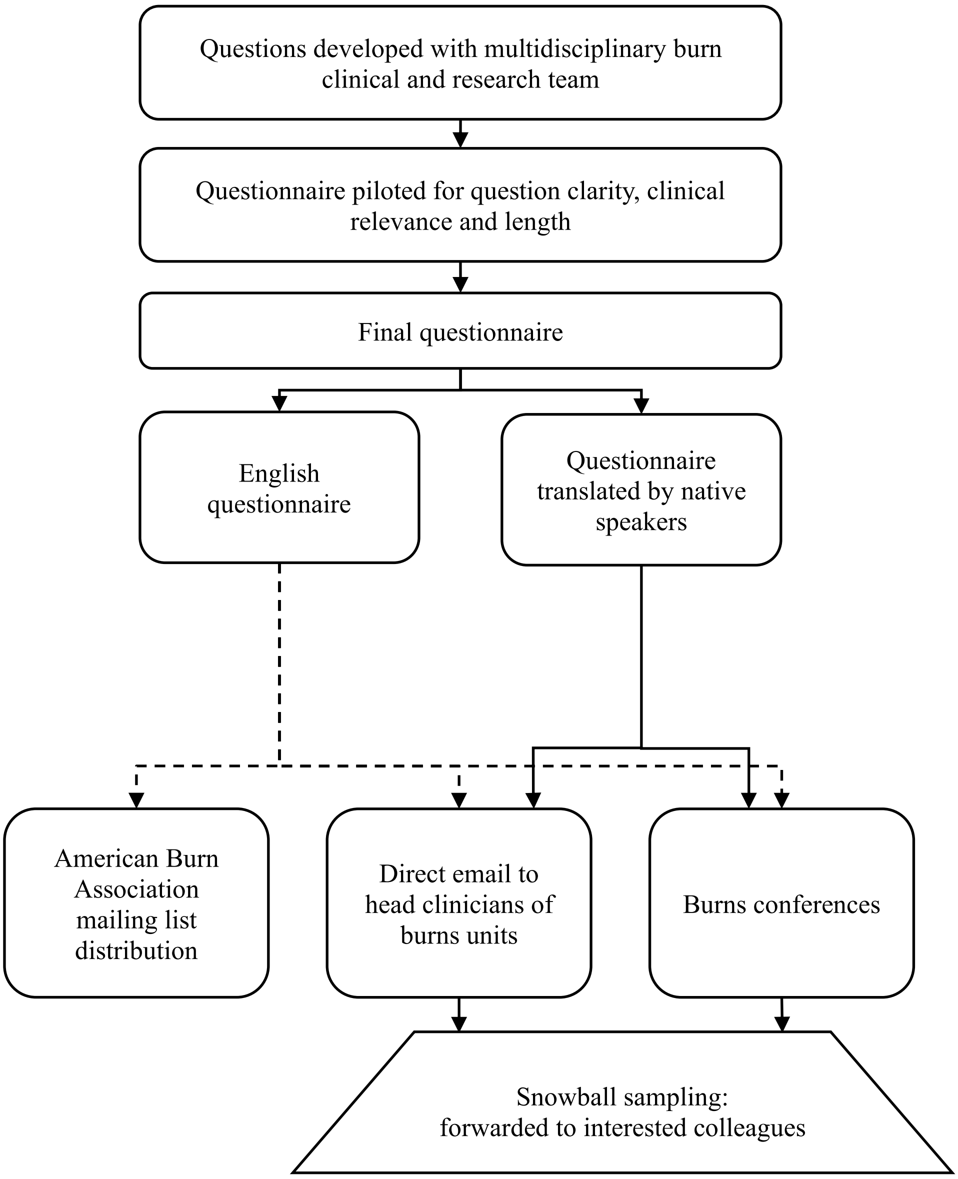

Supplement: Supplementary file 2 — Supplementary Material 2 [file 10103_2026_4919_MOESM2_ESM.pdf]
