# Supplementary material for: Laser treatment of hypertrophic scars: the operative and peri-operative practices of burns clinicians
Source: Lasers Med Sci. 2026 Jun 24;41(1):128. doi: 10.1007/s10103-026-04919-z (PMC13294306; doi:10.1007/s10103-026-04919-z)
Supplement: Supplementary file 3 — Supplementary Material 3 [file 10103_2026_4919_MOESM3_ESM.pdf]

Article title: *Laser treatment of hypertrophic scars: the operative and peri-operative practices of burns clinicians*

Journal title: Lasers in Medical Science

Authors: Maria Shilova, Roy Kimble, Robert S Ware, Karin Plummer, Orlando Flores, Hui (Grace) Xu, Bronwyn Griffin.

Corresponding author: Maria Shilova (School of Nursing and Midwifery, Griffith University; Children's Health Queensland, South Brisbane; Centre for Children's Burns and Trauma Research, Children's Health Queensland, South Brisbane, maria.shilova@griffithuni.edu.au)

Supplementary Material 3

The below table provides further detail of the study methodology according to the Checklist for Reporting Results of Internet E-Surveys (CHERRIES).

CHERRIES item category	CHERRIES item	Description of methodology according to item
Design	Describe survey design	This was a descriptive questionnaire of a convenience sample of Burns clinicians who use laser to treat hypertrophic scars.
Institutional Review Board (IRB) approval and informed consent process	IRB approval	This study received ethical review exemption from the Queensland Children's Hospital Human Research Ethics Committee (EX/21/QCHQ/79011) and approval from the Griffith University Human Research Ethics Committee (2021/828).
	Informed consent	The first page described the purpose of the trial, listed the study investigators, the approximate time required to complete the questionnaire, advised that the data would be stored in a de-identified manner and would be reported in presentations and publications. Participants were informed that progressing with the questionnaire would imply their consent to participate in the research.
	Data protection	Personal data were stored securely on and then on a password-protected computer while data were being cleaned. Personal data were removed from computer files during the data cleaning stage.
Development and Pre-testing	Development and testing	The questionnaire was developed with a multidisciplinary team of three burns clinicians (two Burns consultants and a Burns nurse practitioner) who use laser technology to treat hypertrophic scars.

		The questionnaire was tested for usability and technical functionality.
Recruitment process and description of the sample having access to the questionnaire	Open survey versus closed survey	Participants accessed the questionnaire via web-link or Quick Response code without an entry passcode.
	Contact mode	See Supplementary Material 2.
	Advertising the survey	See Supplementary Material 2.
Survey administration	Web/e-mail	The Griffith Online Research Survey Tool platform (Griffith University, Australia) automatically captures and tabulates responses, which were exported directly for data analysis.
	Context	Not applicable.
	Mandatory/voluntary	Participation was voluntary.
	Incentives	No completion incentives were offered.
	Time/date	December 2021 to June 2023
	Randomization of items or questionnaires	The questions and pages were not randomized
	Adaptive questioning	Adaptive was used (see Supplementary Material 1)
	Number of items	There were 12 main questions, with further questions displayed conditionally based on the respondent's answers to previous questions (see Supplementary Material 1).
	Number of screens (pages)	The questionnaire was distributed over three pages: (1) Demographic data, (2) Procedure details and (3) Post-procedural care.<br><br>The "Procedure details" page had the most questions (up to 10).
	Completeness check	A completeness check was not performed
	Review step	Participants could change their responses while the questionnaire page was open.
Response rates	Unique site visitor	Responses were identified as being unique based on personal details entered on the first question page of the questionnaire.
	View rate (Ratio of unique visitors who agreed to participate/unique first survey page visitors)	A unique visitor view rate was not possible obtain with this questionnaire, due to the way that unique site visitors were recorded.
	Participation rate (ratio of unique visitors who agreed to participate/unique first survey page visitors)	A participation rate was not possible to obtain with this questionnaire, due to the way that unique site visitors were recorded.
	Completion rate (Ratio of users who finished the survey/users who agreed to participate)	A completion rate was not possible to obtain with this questionnaire, due to the way that unique site visitors were recorded.

Presenting multiple entries from the same individual	Duplicates	Duplicate entries were identified by identifying details, after which these identifying details were removed. If a duplicate response was identified, the more complete response was used for analysis
	Cookies used	Cookies were not used
	IP check	IP check was not used
	Log file analysis	Log file analysis was not used
	Registration	Not applicable (open questionnaire)
Analysis	Handling of incomplete questionnaires	Personal information was stored so that participation invitations were not duplicated. These identifiable data were kept during the data cleaning stage to identify duplicate responses and were removed afterwards. Responses recorded as part of testing of the questionnaire were also removed.
	Questionnaires submitted with an atypical timestamp	Not applicable
	Statistical correction	Not applicable
